# Cell-Sized Liposomes and Droplets: Real-World Modeling of Living Cells

**DOI:** 10.3390/ma5112292

**Published:** 2012-11-13

**Authors:** Tsutomu Hamada, Kenichi Yoshikawa

**Affiliations:** 1 School of Materials Science, Japan Advanced Institute of Science and Technology, 1-1, Asahidai, Nomi, Ishikawa 923-1292, Japan; 2 Faculty of Life and Medical Sciences, Doshisha University, 1-3, Tatara Miyakodani, Kyotanabe, Kyoto 610-0394, Japan

**Keywords:** liposome, droplet, cell-sized confinement, lipid membrane, DNA, compartmentalization, nanoparticle

## Abstract

Recent developments in studies concerning cell-sized vesicles, such as liposomes with a lipid bilayer and water-in-oil droplets covered by a lipid monolayer, aim to realize the real-world modeling of living cells. Compartmentalization with a membrane boundary is essential for the organization of living systems. Due to the relatively large surface/volume ratio in microconfinement, the membrane interface influences phenomena related to biological functions. In this article, we mainly focus on the following subjects: (i) conformational transition of biopolymers in a confined space; (ii) molecular association on the membrane surface; and (iii) remote control of cell-sized membrane morphology.

## 1. Introduction

A cell enclosed by a membrane is a common structural unit that is shared by all living organisms. In addition to a plasma membrane, eukaryotic cells have several internal membranes, such as those surrounding the nucleus and organelles. These membranes show a common feature as self-assembled structures of lipid molecules with an amphiphilic nature. The constituent lipids form a bilayer interface with hydrophobic tails oriented toward the interior. A two-dimensional surface consisting of a lipid bilayer embedded in three-dimensional actual space is essential for living cells to maintain their active lives. The µm-scaled vesicular space enclosed by a membrane contains giant DNA molecules above the size of µm that carry genetic information and an enormous quantity of reactions among biomolecules.

Lipid membranes exhibit the following indispensable characteristics for living systems: (i) The membrane interface acts not only as a fence, but also as a molecular sieve by preventing the entrance of “undesired” molecular components while allowing the diffusion of “desired” molecules, by making µm-scaled living environments; and, (ii) lipid membranes are a rather tough soft matter assembled in a spontaneous manner, exhibiting a deformable barrier against thermal agitation and/or external stress. In some occasions necessary to survive, small perturbations in molecular levels can lead to profound changes in mesoscopic organized structures.

The constructive and synthetic approach to biological systems is an important and interesting challenge that spans across several disciplines, including physics, chemistry and biology [1,2]. In this review article, we first introduce cell-sized lipid vesicles as real-world models of living cells, and then describe recent developments regarding cell-sized model systems. We show that, in a cell-sized confined space, the membrane interface influences the conformational transitions of encapsulated biopolymers. Next, we focus on molecular-binding events on a membrane surface with biomimetic heterogeneity. Finally, we demonstrate the experiment on the successful remote-control of membrane morphology, as it relates to cell-sized compartmentalization, manipulated by light. 

## 2. Modeling of Living Cells

### 2.1. Cell-Sized Liposomes

**Figure 1:**
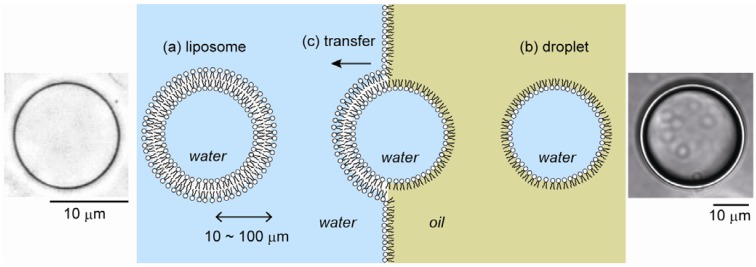
Schematic illustrations and typical microscopic images of cell-sized liposomes (**a**) and droplets (**b**). (**c**) Liposomes can be prepared by the transfer of droplets across an oil/water interface.

The main components of the cytomembrane are phospholipids, cholesterol and membrane proteins. Phospholipids exhibit essentially the same closed bilayer structure (called lipid vesicles or liposomes) as living cell membranes due to amphiphilic properties. Liposomes have been actively studied as a simple model of cell membranes, and also as microreactors for biochemical reactions. They are also widely used as microcapsules in targeted drug delivery and gene therapy in medical practice and in the cosmetics industry because of their affinity for the living body [3]. Liposomes range in size from 50 nm to 10 µm or greater. Currently, numerous studies have been and are performed on submicron liposomes, including on their encapsulation efficiency using fluorescence spectrometry and on membrane structures using electron microscopy or X-ray scattering. Notably, these experiments can only provide ensemble average because of the resolution limit with optical microscopy. Thus, by using such small liposomes, it is almost impossible to trace the time-dependent change in individual liposomes. In addition, these small liposomes are usually not so stable and tend to cause aggregation/fusion because of their high curvature. In contrast, cell-sized liposomes with a diameter of ~10 µm are large enough to allow the real-time manipulation/observation of transformations and encapsulated biochemical reactions in each compartment by optical microscopy. Thus, cell-sized liposomes, which are similar to natural cell structures in terms of size and membrane composition, have attracted considerable attention for the modeling of living cells (Figure 1a) [1,4].

### 2.2. Cell-Sized Droplets

Water-in-oil (W/O) microdroplets coated by phospholipids can also be studied as a model of living cells, since phospholipid molecules are arranged on the surface with their hydrophilic moieties oriented toward the inner aqueous phase, as a plasma membrane (Figure 1b) [5,6]. Water droplets in an oil medium have many advantages over liposomes as model membrane systems. For example, it is difficult to prepare liposomes with physiological aqueous solutions because of their high sensitivity to osmotic or mechanical stress. In contrast, since microdroplets show good resistance to osmotic or physical stress, it becomes possible to conduct experiments under physiological ionic conditions, and biological macromolecules can be easily encapsulated without being denatured. Furthermore, droplets can be used to prepare liposomes [7]. Water droplets with a lipid monolayer, intended for the inner leaflet, produce vesicles by passing through a second oil–water interface, where they become coated with the outer leaflet (Figure 1c) [8]. Notably, a method with two independently prepared monolayers has enabled the formation of asymmetric vesicles with hybrid bilayer structures between the inner and outer leaflets, like cytomembranes [9,10].

## 3. Encapsulation of Biopolymers in a Cell-Sized Confined Space

Through the usage of droplets, it has become possible to encapsulate desired amounts of macromolecules, such as genomic DNA, proteins, and various kinds of hydrophobic polymers, under well-controlled solution conditions, including pH, salt concentration, *etc.* In a small cell-sized space, the surface/volume ratio increases, which suggests that lipid membrane surfaces may have an important effect on cellular chemical reactions. To understand the characteristics and driving forces of the molecular events that occur within a cell, studies under conditions of cell-sized confinement, in addition to those in a bulk system, are needed.

### 3.1. Conformational Transition of DNA in a Confined Space

In a cell, DNA and other molecules are present in a micrometer-scale confined space. It is known that a long DNA molecule, above the size of several tens of kilo base pairs (kbp), exhibits a large discrete conformational change from a coiled state to a highly folded state in aqueous solution, depending on the presence of various condensing agents such as polyamines [11,12]. To elucidate the characteristics of the conformational behavior of DNA molecules in a confined space, a large DNA (bacteriophage T4DNA of 166 kbp labeled with fluorescent dyes) was encapsulated in a droplet [13].

**Figure 2:**
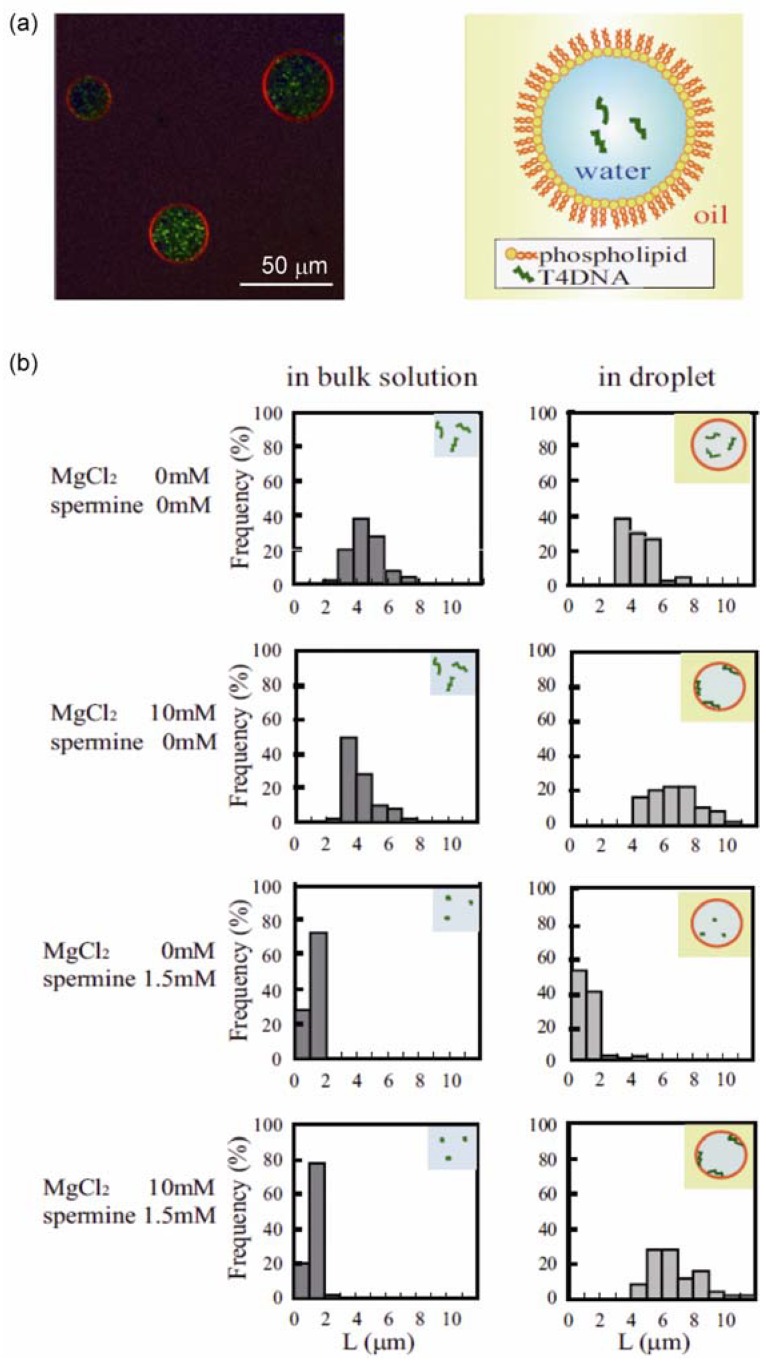
(**a**) Microscopic image of cell-sized droplets encapsulating T4 DNA, together with schematics (right); (**b**) Histogram of the long-axis length (*L*) of T4 DNA molecules under various conditions. Reprinted from [13]. Copyright 2009 with permission from Elsevier.

Figure 2a shows confocal laser scanning microscopy images of the prepared droplets. Red indicates a fluorescent lipid analog, TRITC-DHPE, and green indicates YOYO-1, which bound to T4DNA molecules. The images show that our preparation made droplets with diameters of mostly 20 to 60 µm, each of which was coated with thin phospholipid layers, and these encapsulated T4 DNA molecules. First, we investigated the distribution of T4 DNA molecules within a microdroplet (Figure 2b). In a droplet composed of dioleoyl phosphoethanolamine (DOPE), DNA molecules were diffusely distributed in the aqueous phase of the droplet in the absence of Mg^2+^. When Mg^2+^ was present at 10 mM, almost all of the DNA molecules were located on the inner surface of the droplet (membrane surface). The localization of DNA molecules on the membrane surface in the presence of Mg^2+^ is a phospholipid headgroup-dependent phenomenon. When the droplets were prepared with egg phosphatidylcholine (eggPC) instead of DOPE, T4 DNA molecules were distributed in the aqueous phase and were not bound to the membrane surface, even in the presence of 10 mM Mg^2+^.

Polyamines such as spermidine and spermine induce a conformational transition of long DNA molecules from a coil state to a highly compact folded state in aqueous solution, accompanied by the drastic change of the segment density on the order of 10^4^–10^5^ [14]. In the buffered solution examined in this study (10 mM Tris-HCl, pH 7.4, 100 mM KCl), all of the T4 DNA molecules showed a highly compact conformation in 1.5 mM spermine. DNA with a compact (folded) conformation was encapsulated into a DOPE microdroplet, and the distribution and conformation of DNA molecules in the droplet were investigated (Figure 2b). In the absence of Mg^2+^, T4 DNAs were mainly located in the aqueous phase with a compact conformation. In the presence of 10 mM Mg^2+^ with 1.5 mM spermine, in the bulk solution, the DNA molecules had a highly compact and folded conformation. When these DNA molecules were encapsulated within a DOPE droplet, most of them were located on the membrane surface. These results show that a DNA molecule with a compact conformation in the bulk solution unfolds its structure through interplay with the phospholipid membrane surface when encapsulated within cell-sized confinement.

In addition, DNA molecules entrapped in large droplets tended to not be adsorbed on the membrane. When the droplet size was <60 µm in diameter, almost all of the DNA molecules were adsorbed onto the membrane surface. When the diameter became larger than 60 µm, some DNA molecules were adsorbed on droplets and others were in the aqueous phase with a folded conformation. A thermodynamic analysis [14] suggested that the loss of translational entropy of a DNA molecule that accompanies adsorption is a key factor in these phenomena under micrometer-scale confinement. When the loss of translational entropy due to adsorption, *i.e.*, from a state confined in three-dimensional space to that on a two-dimensional surface, is comparable to the gain in free energy due to the interaction between DNA and membranes, a small difference in the electrostatic properties of the membrane surface and/or confinement volume should determine whether or not adsorption takes place. The experimental finding that DNA molecules existing within larger droplets tend to be released into the aqueous phase suggests that the interplay between membranes and DNA plays a significant role on the structure and function of genomic DNA in living cells.

These results indicate that biochemical reactions involving DNA, such as gene expression, in a cellular space are possibly different from those in a bulk space, such as in test tubes in a lab. It has been demonstrated that the on–off switching of transcription was regulated by the conformational transition between a folded state in an aqueous phase and an extended coil state on a droplet membrane surface [15]. GFP expression within cell-sized liposomes was reported to be higher than that in bulk solution [16]. With the use of these cell-sized droplet systems, the rate of protein production was shown to be inversely proportional to the radius of the droplets [17]. The acceleration rate depended on the type of covering lipid. Hereafter, it may be of scientific value to unveil the effect of the membrane surface on biochemical systems in living cells.

### 3.2. Structural Transition of Actin Filament in a Confined Space

Actin filament, *F*-actin, is a semiflexible polymer with a negative charge, and is one of the main constituents of cell membranes. It is important to clarify the crosstalk between a phospholipid membrane and *F*-actins inside a cell space. We conducted microscopic observations on the structural changes in *F*-actins in a cell-sized droplet coated with a phospholipid membrane, such as phosphatidylserine (PS) with a negatively charged head group, as a simple model of a living cell membrane [18]. The adsorption–desorption of *F*-actins on the droplet surface was shown to depend on the Mg^2+^ ion concentration. In the absence of Mg^2+^, actin monomers (*G*-actin) were uniformly distributed in a droplet. At 2 mM Mg^2+^, *F*-actins formed and were uniformly distributed in the water phase without adsorption on the membrane surface. As the Mg^2+^ concentration increased below 12 mM, *F*-actins tended to adsorb onto the membrane. At Mg^2+^ concentrations higher than 12 mM, bundles of *F*-actins formed in the water phase, and the number of actin filaments attached to the membrane decreased. This indicates that, under cell-sized confinement, interaction with lipid membranes induces a unique structure of actin filaments such as a cortex shell. Furthermore, Takiguchi *et al.* have developed cell-sized liposomes that encapsulate heavy meromyosin (HMM)/*F*-actin complexes by the transfer of droplets [19]. A critical vesicle size was found to determine the structure of the encapsulated *F*-actins. *F*-actins localized around the inner periphery of liposomes smaller than the critical size, whereas in the bulk solution and in larger liposomes, *F*-actins formed aster-like structures under the same conditions. Recently, several studies have also described the reconstitution of cytoskeletons in cell-sized compartments [20,21,22].

## 4. Association on Soft Membranes

### 4.1. Microdomains

Plasma membranes are not just a surface in which proteins are embedded, but rather dynamically organize their interfacial structures. Within membranes, microdomains called lipid rafts are formed to effectively produce lateral compartments with a high lipid order and slow dynamics [23]. Bilayer lateral heterogeneity is considered to be a form of order-disorder phase separation that develops due to the interaction between membrane lipids. The organized domains are expected to play an important role in the selective associations of materials during molecular-binding events, such as signaling or toxic processes [24]. This indicates that the mechanical properties of membranes, such as lateral fluidic heterogeneity, are coupled to functional molecular recognition within the membrane surface. Microscopic domain structures due to order–disorder phase separation have been observed in cell-sized liposomes that are simply composed of saturated and unsaturated lipids together with cholesterol [25]. The equilibrium phase diagram of ternary lipid systems has been well characterized, and the membrane phase can mainly be classified into three states: liquid-disorder (Ld), liquid-order (Lo) and solid-order (So) [26,27]. Studies with these simple model membrane systems are invaluable for elucidating how molecules and materials interact with heterogeneous membranes. Recently, such membrane systems have been used to study Lo/Ld phase-partitioning for several signaling proteins and their membrane anchors [28].

### 4.2. Size-Dependent Partitioning of Nanoparticles

**Figure 3:**
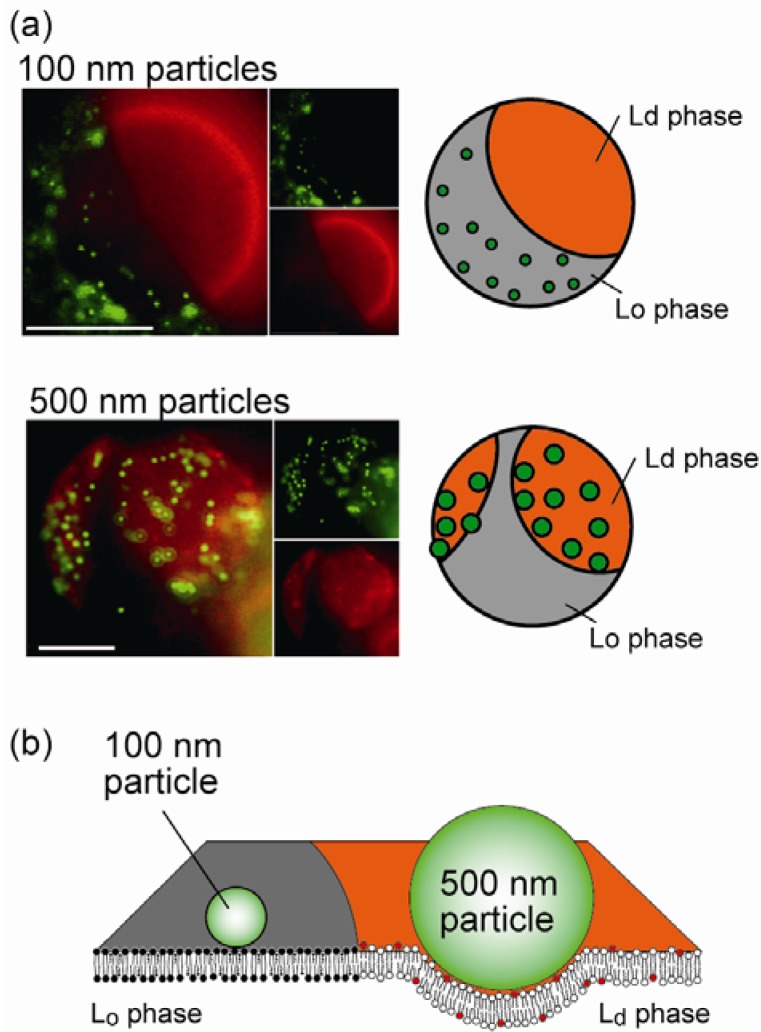
(**a**) Typical images of the localization of nanoparticles (NPs) with diameters of 100 and 500 nm on heterogeneous bilayer membranes. Scale bars are 10 µm; (**b**) Schematics of the size-dependent selective localization of NPs. Reprinted with permission from [33]. Copyright 2010 American Chemical Society.

Over the past decade, nanoscience and nanotechnology have been the focus of increasing interest to achieve advances in functional nanomaterials, such as the application of nanoparticles (NPs) to biological systems [29]. However, the cytotoxic effects of NPs are not well understood and need to be elucidated to ensure safe handling and engineering [30]. It is important that we understand the physical mechanisms that govern the interaction between NPs and the cell surface, which is a soft interface ~5 nm thick with a lipid bilayer. Several studies on membrane–colloid interactions with homogeneous cell-sized liposomes have reported that polystyrene particles spontaneously adhere to a phosphatidylcholine (PC) membrane surface [31,32]. We investigated the association of polystyrene NPs within biomimetic membrane interfaces with lateral heterogeneity, such as two-phase liposomes [33]. Two-liquid Lo/Ld separated liposomes were composed of saturated and unsaturated PC lipids (dipalmitoyl phosphatidylcholine, DPPC, and dioleoyl phosphatidylcholine, DOPC) together with cholesterol (Chol) (DOPC/DPPC/Chol = 37:37:26, molar ratio). The membrane contained a red-fluorescent lipid, rho-PE, which preferentially partitioned into the Ld phase. Green-fluorescent NPs were used to monitor its location. Figure 3a shows typical fluorescent images of liposome surfaces that were simultaneously stained with rho-PE and NPs. NPs localized in a particular membrane phase depending on their sizes (Figure 3b). Smaller NPs of ≤200 nm were localized in the Lo region, while larger NPs of ≥200 nm were partitioned into the Ld region. This partitioning of NPs was reproduced during the mixing/demixing miscibility transition, indicating that this behavior of NPs is mediated by the thermodynamic characteristics of the lipid membranes.

This association preference can be explained by considering the membrane elastic energy with the fluidic difference between two coexisting phases. When a particle strongly adheres to a membrane surface, the membrane curves around the particle’s periphery. The threshold particle radius r* for membrane deformation to wrap a particle is estimated to be r* ≈ (2*κ*/*w*)^1/2^, where *w* is the adhesion energy (J m^−2^), *κ* is the bending stiffness of the membrane (J), and the spontaneous curvature of the membrane is zero. If we assume that the adhesion energy is essentially derived from the van der Waals interaction between the surfaces of a polystyrene and lipid, the adhesion energy can be expressed as *w* = *A*/12π*D*^2^, where *A* is the Hamaker constant, and *D* is the distance between the surfaces of the particle and membrane. We obtain r^*^ = 130 nm (260 nm diameter), which is close to the experimentally obtained threshold diameter (200 nm). This indicates that the degree of membrane deformation induced by NP adhesion may determine the partitioning phase of NPs. The adhesion of larger NPs of r > r* tends to induce the deformation of membrane curvature, while smaller NPs of r < r* do not alter such mesoscopic membrane structures. When a membrane exhibits bending deformation due to the association of large particles, the free energy cost per unit area can be written as $\Delta F=\frac{\kappa }{2}{\left(c_{1}+c_{2}-c_{0}\right)}^{2}$ where c_1_ and c_2_ are the two principal curvatures, and c_0_ is the spontaneous curvature. Since the free energy is proportional to *κ*, a difference in bending stiffness between the Ld and Lo phases (*κ*_Ld_ < *κ*_Lo_) leads to ∆*F*_Ld_ < ∆*F*_Lo_, indicating that large particles of *r* > r^*^ tend to distribute in the Ld phase of Lo/Ld membranes. In contrast, for small particles of *r* < r^*^, the effect of bending deformation is insignificant. However, when particles are situated close to the membrane surface, thermal undulation of the membrane should be restricted in a finite space. For the adhesion of small particles of *r* < r^*^, the change in membrane undulation may be considered instead of bending deformation. The free energy cost per unit area for restricted undulation is expressed as $\Delta F=\frac{3{\left(k_{B}T\right)}^{2}}{2\pi^{2}\kappa }\frac{1}{d^{2}}$ , where *d* is the length of the space. The free energy is inversely proportional to *κ*, indicating that ∆*F*_Ld_ > ∆*F*_Lo_, which explains why smaller particles tend to localize in the Lo phase while larger particles partition into the Ld phase, in agreement with our experimental results.

These findings strongly indicate that not only specific molecular interactions, such as those of receptor proteins, but also the mechanical properties of the fluid bilayer itself play an important role in the selection of associating materials into lateral compartments. Cells may take advantage of the thermodynamic characteristics of the membrane to recognize contacting materials and/or to distribute their biomolecules. Notably, biological raft domains are expected to function as platforms that form such endocytic carriers [34], and endocytosis-like transformation has been demonstrated using a cell-sized model system [35]. We are currently investigating the dynamical behavior of nanoparticle-membrane systems, such as endocytosis, since nanomaterials that are in contact with plasma membranes are eventually engulfed by wrapping.

### 4.3. Selective Localization of Peptides

The lateral localization of molecules profoundly influences the cellular response. As an example, we describe Alzheimer’s amyloid β peptide (Aβ). Aβ has attracted much attention as one of these membrane-associating proteins because of its role in the pathology of Alzheimer’s disease [36]. Aβ peptides with 40 or 42 amino acid residues, Ab-40 or -42, are produced from an amyloid precursor protein embedded in plasma membranes. Aβ monomers aggregate into fibrils via oligomers, leading to the formation of senile (amyloid) plaques in the brains of patients with Alzheimer’s disease [37,38]. The mechanism of the association of Aβ on a cell membrane surface is poorly understood and needs to be elucidated.

We can use phase-separated cell-sized liposomes to detect the localization of Aβ-42 aggregation species [39]. As in the association of NPs, lateral heterogeneity of the membrane was found to mediate the localization of Aβ-42 in a peptide aggregation-dependent manner. For Lo/Ld separated membranes, oligomers and prefibrils of Ab-42 were partitioned in the Ld phase, whereas fibrils did not localize in the membrane but rather floated in an aqueous solution. When we decreased the Chol fraction to induce So/Ld separation, fibrils adsorbed on the membranes and showed a partitioning preference into the So phase. This indicates that the mechanical properties of the membrane play an important role in the interaction with membrane-associating peptides. A change in the fluidity of membrane domains may be a key factor in the onset of Alzheimer’s disease.

## 5. Transformable Boundary for Cell-Sized Compartments

Living cells dynamically organize their membrane compartments to exhibit cellular functions, such as vesicular trafficking. A better understanding of the physical mechanism that underlies such soft membrane dynamics could lead to the manipulation of artificial cellular compartments. The stability of lipid bilayer compartments is determined by the elastic free energy of the membranes, which is given by the sum of the bending and line energies $F=\int \left[\frac{\kappa }{2}{\left(c_{1}+c_{2}-c_{0}\right)}^{2}+\kappa^{\prime }c_{1}c_{2}\right]dA+\gamma \int dl$ , where *dA* is the area element of the membrane surface, *dl* is the line element along the membrane pore, *c*_1_ and *c*_2_ are the two principal curvatures, *c*_0_ is the spontaneous curvature, *κ* is the bending modulus, *κ*' is the Gaussian bending modulus, and *γ* is line tension. Within bilayer membranes, edges are disfavored because of the high energy cost of exposing the hydrophobic lipid chains to water. Thus, *open* membranes with either an edge or a pore have the line energy, *i.e.*, an energy cost per unit length of the exposed edge. For 10 µm-scale membranes, which are the typical size of cells, a *closed* membrane without a boundary is energetically more stable than the *open* form, since the cost of the bending energy to obtain a *closed* membrane (~10^−19^ J) is much lower than that of the line energy needed to expose the bilayer rim of the *open* membrane (~10^−17^ J). This indicates that a decrease in line tension induces a stable pore within bilayer vesicles. Indeed, a membrane pore was experimentally observed in model membrane systems only upon treatment with additives such as surfactants [40,41] or proteins [42].

**Figure 4:**
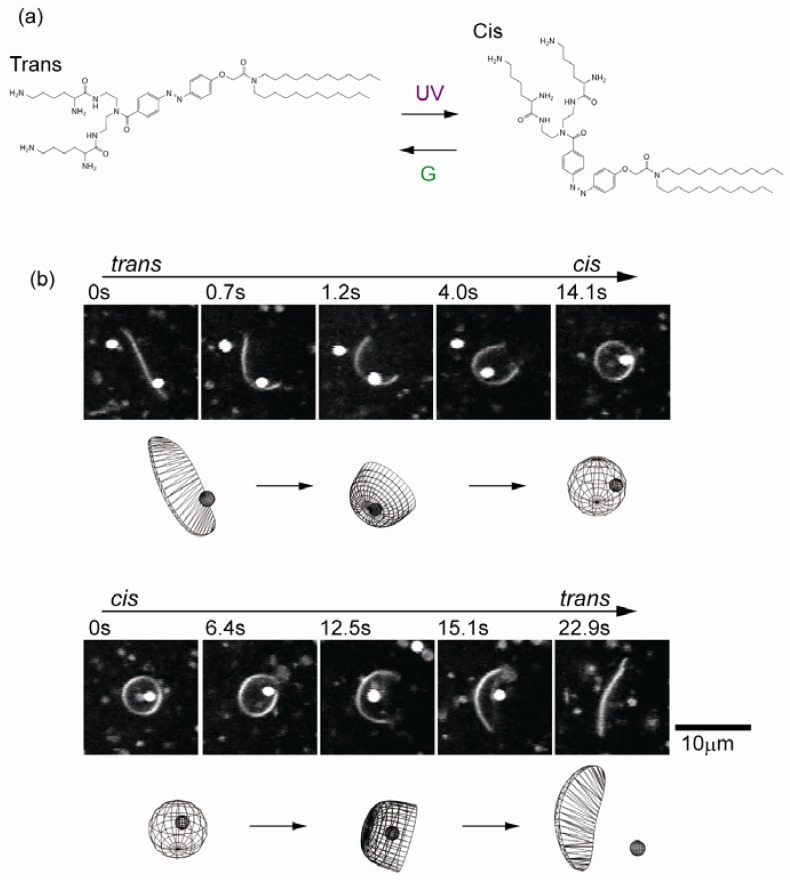
(**a**) Structural formula of the azobenzene-containing amphiphile (KAON12); (**b**) Reversible formation of a cell-sized vesicular compartment. The capture and release of a colloidal particle (500 nm). Reprinted with permission from [46]. Copyright 2010 American Chemical Society.

Through the use of a synthetic photosensitive amphiphile containing an azobenzene unit (KAON12) (Figure 4a) [43,44,45], we succeeded in controlling the membrane to give *open* sphere and *closed* disc shapes in which the shape of a vesicle can be switched by light [46]. The membranes are prepared from a binary mixture of KAON12 and a phospholipid, dioleoyl phosphatidylcholine (DOPC), with Tris-HCl (pH 7.4) buffer solution. The photoisomerization of KAON12 from the *trans*- to *cis*-form transformed the *open* membrane disc into a *closed* spherical structure. The *closed* sphere reverted to an *open* disc under *trans*-isomerization. This membrane system enables the capture and release of targeted objects, such as in vesicular transport. Figure 4b demonstrates the reversible capture and release of a colloidal particle (0.5 µm) into and from the membrane. During the transition from the open to closed dynamics upon UV irradiation, a colloidal particle was captured into the *closed* vesicular space. Release of the particle from the membrane sphere was achieved by *trans*-isomerization.

This morphological transition can be attributed to the change in membrane elastic energy upon photoisomerization. We analyzed the thermal motion of the edges of membrane discs. The fluctuation of an edge line is related to the line energy of the membrane, since the line energy is proportional to the length of the exposed edge. We found that the discs showed a marked difference in membrane edge fluctuation between isomers; fluctuation of the *trans*-membrane edge was greater than that of the *cis*-membrane edge. The relationship between the Fourier coefficients and wave number and line tension is given by $\langle a_{k}^{2}\rangle +\langle b_{k}^{2}\rangle =\frac{2k_{B}T}{\pi r_{0}\gamma }\frac{1}{k^{2}-1}$ , where *a_k_* and *b_k_* are the Fourier coefficients, *k_B_* is the Boltzmann constant and *T* is the absolute temperature [47]. The analysis of the membrane edge fluctuation revealed that the line tension of *trans*-membranes (*γ*_trans_ = 5.0 ± 2.2 × 10^−2^ pN) was smaller than that of *cis*-membranes (*γ*_cis_ = 1.5 ± 0.4 × 10^−1^ pN). Photoisomerization caused switching of the interfacial line tension of membranes. For a membrane sphere with a radius of 10 µm, a critical line tension for compartmentalization is estimated to be *γ*_c_ = 7.2 × 10^−2^ pN based on a simply comparison of the free energies of a sphere (12*πκ*) and disc (4*πγR*_0_). Photoisomerization switches the line tension between above and below the critical value (*γ*_trans_ < *γ*_c_ < *γ*_cis_).

## 6. Concluding Remarks

This review described recent experimental developments regarding cell-sized droplets and liposomes with the aim of better understanding the physical mechanisms that govern the organization of living cell systems. It has been shown that, due to the large surface/volume ratio in the micro space, encapsulated biopolymers exhibit unique self-organized structures through interplay with membrane surfaces. The soft-matter characteristics of the membranes play an important role in the association of molecules and the formation of compartment capsule structures. Along these lines, several recent studies have described cell-sized model systems, such as a model that focused on a high density of macromolecules within cells [48,49,50,51]. Notably, membrane compartment systems have also been reported to show a simple selection or competition process within vesicle populations as a primitive demonstration of the characteristics of living things [52]. As the future research target, it may be useful to explore membrane characteristics under nonequilibrium conditions, since dynamic phenomena concerning lipid membranes in living organisms such as vesicular transport, division and growth are time-dependent processes. Recently, some experimental trials have examined membrane behaviors under nonequilibrium conditions, such as a change in tension by active proteins [53] and the oscillatory motions of membrane pores [41,54]. Further studies on the dynamical motions of cell-sized compartment systems that are out of equilibrium are awaited.
